# Sex Differences in Sexual Motivation in Humans and Other Mammals: The Role of Conscious and Unconscious Processes

**DOI:** 10.3390/bs14040277

**Published:** 2024-03-27

**Authors:** Priscille Touraille, Anders Ågmo

**Affiliations:** 1 Centre National de la Recherche Scientifique (UMR 7206), Muséum National d’Histoire Naturelle, 75116 Paris, France; priscille.touraille@mnhn.fr; 2 Department of Psychology, University of Tromsø, 9037 Tromsø, Norway

**Keywords:** sexual scripts, genital response, human, primate, rodent, implicit motivation, automatic (unconscious) responses, volitional responses

## Abstract

In self-report questionnaires, men report higher scores than women on variables such as desire for sex, frequency of sexual thoughts, number of sex partners, etc. Based on this, men are considered to have a higher level of sexual motivation than women. However, retrospective self-reports may be unsuitable for estimations of the inherent level of sexual motivation. We review data on automatic (unconsciously controlled) responses and measures of implicit motivation during exposure to sexual stimuli. These responses and measures are inaccessible to willful manipulations and make it possible to determine whether the sex difference in answers to questionnaires is replicated when volitional response manipulations are unlikely. We complement the human data with observations from some rodent and non-human primate species. The attentional resources allotted to stimuli with sexual relevance as well as genital responses to such stimuli are similar in men and women. Measures of implicit motivation also fail to detect any sex difference. Finally, the frequency of masturbation is superior in female infants before the age at which social expectations begin to determine behavior. Neither in rodents nor in non-human primates is there any clear-cut evidence for sex differences in motivation. It seems that males and females are similar with regard to the intensity of sexual motivation. The responses to questionnaires may be affected by social learning of sexual scripts and/or the inferior quality of sexual experiences in women, among other things.

## 1. Introduction

In popular culture, it is often stated that men have a higher level of sexual motivation than women (e.g., [1]). This point of view seems to have considerable factual support. A review of the relevant literature [2] found that men systematically reported a higher frequency of sexual thoughts, a larger number of desired sexual partners, and a higher frequency of desire for intercourse than women. In fact, regardless of the measure used as an indicator of sexual motivation, it was found that men scored higher than women did. It was concluded that men indeed have a higher sexual motivation than women and that the sex difference is caused by a mixture of biological and social factors. A similar conclusion was reached in an extensive survey with participants from many countries [3]. A meta-analysis based on 211 studies reporting on sex differences in sexual motivation confirmed that men think and fantasize about sex more often than women do. Likewise, men experience sexual desire and masturbate more frequently than women do. Neither response bias nor any other moderator variable could account for the differences. These observations were interpreted as evidence of a higher level of sexual motivation in men than in women [4]. 

All the data supporting the existence of sex differences in sexual motivation mentioned in the preceding paragraph stem from self-report questionnaires of different types. However, it has been pointed out that such questionnaires are notoriously unreliable when it comes to surveys of sexual behaviors [5]. There are several reasons for this. One is that responses may be heavily influenced by social desirability and other irrelevant factors. In a much-cited study, the participants were either made to believe that untrue responses would be detected because they were connected to a lie detector, that their responses would remain anonymous, or that the experimenter could view their responses [6]. In the first condition, the sex difference was negligible, whereas it was considerable in the third condition. The difference between conditions was particularly large for behaviors considered less acceptable for women, for example, masturbation or pornography consumption. Similar data were obtained in a study of gaze patterns in men and women exposed to pornographic stimuli [7]. When participants believed that their gaze was monitored, there was a clear sex difference. When not, this difference disappeared. It was concluded that women suppressed their sexual interest during implied social presence. In an internet survey, it was found that assurance of anonymity had a large effect on women’s responses but less on men’s responses [8]. When young men and women were exposed to a pornographic movie segment while their genital responses as well as their subjective sexual arousal were reported it was found that the genital response was unaffected by the presence of equipment for lie detection. Moreover, the correlation between the self-reported subjective sexual arousal and the genital response was enhanced in women in the lie detection condition. The rather complex results were interpreted as showing that gender norm conformity affects sexual response patterns, especially in women [9]. All these observations may be related to the fact that women are taught to suppress their sexuality, making them recur to lies about sexual issues more frequently than men (e.g., [10]). An overview of how social desirability distorts responses to sensitive questions as well as some ways to reduce its impact is available [11]. Whether any of these ways have been employed in studies of sex differences in sexual motivation is questionable.

Response bias is not the only problem with self-reports. For obvious reasons, such reports are limited to conscious experiences. However, parts of sexual motivation remain unconscious [12,13,14] or implicit if that term is preferred [15], and these parts will inevitably be ignored in self-reports. Thus, such reports will only reflect a fraction of the sexual motivation. In addition to problems with social desirability and the omission of the unconscious elements of motivation, self-reports suffer from other potential sources of unreliability, such as recall bias, retrospective bias, low literacy among respondents, and poor comprehension of sexual behavior questions [16]. The latter sources of unreliability may become of particular importance in cross-cultural studies (e.g., [3]). Because of the inherent weaknesses of self-reports, any analysis of sex differences based on such reports must be interpreted with caution. 

Instead of estimating the intensity of sexual motivation through retrospective self-reports, direct measurements can be made. There are several ways to record manifestations of sexual motivation in real time without any need to probe into memories by means of questionnaires. The purpose of the present contribution is to review the evidence in favor of and against the existence of an inherent sex difference in the intensity of sexual motivation as determined by direct observation of behavioral and visceral manifestations of sexual motivation. We will also review the recent studies assessing implicit sexual motivation in humans, an approach encompassing both conscious and unconscious elements. 

The specific kind of manifestations to be recorded and quantified as well as the procedures used for activating sexual motivation are determined by the motivational theory on which the study is based. Therefore, it is necessary to provide a brief overview of the different theoretical approaches to the study of sexual motivation. We will conclude that the incentive motivation framework not only has become dominant in studies of sex behavior and motivation, but it is also the most appropriate basis for evaluating sex differences. We will also present a short overview of the methods used for the quantification of sexual motivation, including the recent efforts to develop new methods including the unconscious contribution to the momentaneous level of that motive. 

Finally, we will examine data regarding differences between males and females. In this context, we will summarize observations suggesting that women experience less enjoyment of sexual activities than men do. The main factors contributing to this difference will be discussed, and we will suggest that the inferior quality of women’s sexual encounters is a viable explanation for self-reported sex differences in motivation. 

Throughout this review, we will provide relevant data from non-human mammals in addition to human data. Direct quantifications of sexual motivation in males and females are possible in rodents, allowing for highly precise sex comparisons, and indirect data are available from primates. Since there is no reason to believe that the basic mechanism of sexual motivation is any different between human and non-human mammals, sex comparisons in non-human mammals are highly relevant to the question of inherent sex differences in sexual motivation.

## 2. Approaches to Sexual Motivation

### 2.1. Instinct Theories

There are many approaches to the abstract concept of sexual motivation. Essentially, scientists have been concerned with two basic issues: The intimate nature of sexual motivation itself and the behavioral manifestations of this motive. Among the pioneers, Albert Moll [17] considered sexual motivation as an inherited instinct, consisting of two elements, the drive for contrectation (Contrectationstrieb) and the drive for detumescence (Detumescenztrieb). The former is a drive to approach, touch, and kiss another individual, associated with tumescence. It may also be associated with emotions such as closeness or love. Detumescenztrieb is a drive for copulation, ending in ejaculation and orgasm. This leads eventually to detumescence in the male. In women, orgasm leads to a corresponding reduction in genital blood volume. In both sexes, approach and the ensuing tactile stimulation as well as copulation, particularly orgasm, give rise to voluptuous experiences. 

Another instinct theorist, William McDougall, conceived the inborn sexual instinct as having three elements; cognitive, affective, and conative [18]. The cognitive and affective elements refer to the knowledge of an object that can satisfy the instinct and the affective component is the emotion that the object arouses in the organism. Conation refers to the mental processes of volition, acts of will, leading to the striving toward a goal. Thus, the conative part will eventually lead to the voluntary motor activities needed for the fulfillment of the instinct. It is important to note that Moll as well as McDougall considered that the sexual instinct is activated by stimuli with sexual significance, provided that the nervous system is in a state that makes it responsive to such stimuli. This becomes particularly clear in McDougall’s definition of the sexual instinct [19].

These instinct theories were influential during the first couple of decades of the previous century but are now largely forgotten. Nevertheless, some of their basic assumptions have been incorporated into some contemporary theories of sexual motivation. A somewhat different approach was outlined by Sigmund Freud [20]. His groundbreaking analysis of the nature of the sexual drive [21] has still considerable influence in some circles. A summary of the early theories of sexual motivation is found elsewhere [12]. It is most unlikely that any of the instinct-based approaches to sexual motivation can contribute neither to establishing nor to elucidating potential sex differences.

### 2.2. Sexual Economics Theory

A model related to the old instinct theories but in a different guise, sexual economics theory (SET) coincides with the sociobiological notions of female choosiness and male promiscuity. Sex is regarded as a commodity, and the model proposes that sexual interactions are directed by market forces, in which women are sellers and men are buyers [22,23]. A basic premise for this model is that men have a higher level of sexual motivation than women. Indeed, “gender differences in sex drive are, therefore, a fundamental premise rather than a prediction of SET, and considerable theoretical revision would be needed should it turn out that there is no such gender difference” [4] (p. 628). This approach to sexual motivation has been well-received among conservative men, particularly those convinced of male supremacy [24]. It would not be appropriate for testing the very notion on which it is based.

### 2.3. Self-Determination Theory

Among the contemporary theoretical approaches used for understanding sexual motivation, the self-determination theory proposed by Deci and Ryan [25] may be one of the most elaborated. It represents a connection with instinct theory because a series of innate, psychological needs are at the basis. The three needs are autonomy, competence, and relatedness. Briefly, the theory states that optimal functioning depends on the degree to which an individual’s behavior is self-determined, i.e., behavior is autonomous. The highest level of self-determination is achieved when motivation is intrinsic, meaning that the individual engages in a particular behavior because the behavior itself is rewarding. The lowest level of self-determination is found when the individual performs activities because of external pressures from others or to obtain an external reward. Instead of being autonomous, behavior is controlled by external forces. Amotivation is a complete absence of external as well as internal motivation, and acts are performed without any intention. Self-determination theory has provided the basis for the development of a questionnaire for quantifying intrinsic as well as extrinsic sexual motivation [26]. It has been found that autonomous (intrinsic) motivation for engaging in sex is positively associated with sexual and relationship satisfaction as well as with positive sexual affect [27,28], supporting predictions of self-determination theory. This approach to motivation has not been applied to the issue of sex differences in the level of sexual motivation and may not be the most obvious way to address that question [29]. Nevertheless, the theory has inspired research on other kinds of sex differences, e.g., in motivation for physical activity [30,31] or energy intake [32]. It is doubtful whether animals belonging to species other than human are much concerned with the degree of relatedness, competence, or autonomy, at least not as defined in the theory: “Autonomy refers to volition—the organismic desire to self-organize experience and behavior and to have activity be concordant with one’s integrated sense of self” [25] (p. 231). It is most unlikely that concerns about the sense of self play a major role in non-human animals, making self-determination theory unsuitable for any analysis of inherent sex differences in non-human sexual motivation. 

### 2.4. Incentive Motivation Theories

**Figure 1:**
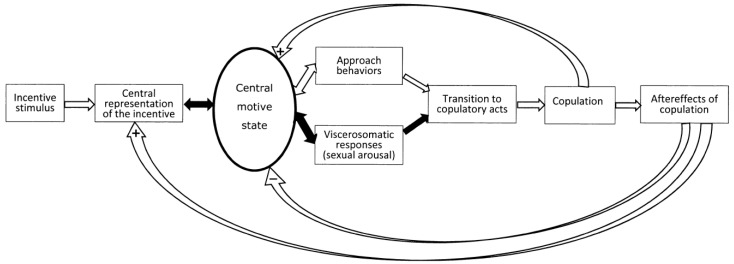
Schematic illustration of the workings of sexual motivation. For explanation, see text. Open, straight arrows illustrate conscious processes in which cognitive evaluations may intervene whereas black arrows indicate automatic (unconscious processes). Curved arrows represent feedback systems. +. stimulation; −, inhibition. Reprinted from [13].

The sexual incentive motivation model originates from the work of Dalbir Bindra [33,34] and its subsequent adaptation to sexual interactions [13,35,36,37]. An excellent account of the wider history of incentive motivation theories is found in [38]. The basic postulate is that sexual motivation is activated by sexual incentive stimuli, either physical in the environment or mental representations of such stimuli. Provided that the nervous system is responsive to these stimuli, sexual motivation will be activated. The hypothetical nervous processes determining the magnitude of sexual motivation are labeled the central motive state. Cognitive processes determine both whether a stimulus will be attributed sexual relevance, hence acting as an incentive, and whether the context is appropriate for allowing the motivational arousal to lead to the display of sexual approach behaviors and eventually copulation [13,39,40]. Another consequence of activation of the central motive state is stimulation of a series of visceral responses, such as enhanced heart and respiration rate, altered galvanic skin response, release of adrenal hormones, and enhancement of genital blood flow. Many of the visceral responses to sexually relevant stimuli are not specific to this particular kind of stimuli. Rather they represent enhanced general arousal, regardless of the source of arousal enhancement. This is the case for the increased heart rate, altered psychogalvanic skin response, pupil dilation, and the release of some hormones (reviewed in [12]). A schematic drawing of the sequence of responses and of their motivational control after exposure to a sexual incentive is found in Figure 1.

The increase in vaginal blood volume and penile erection are specific to sexually relevant stimuli and unrelated to the level of general arousal [41,42]. Moreover, the magnitude of the genital response is dependent on the intensity of the sexual stimulus both in men [43,44] and women [45]. Thus, it can be maintained that the genital response to sexual incentive stimuli reflects the activity in the sexual central motive state. Recordings of genital responses can, therefore, provide reliable and unbiased information concerning the intensity of sexual motivation. The enhanced blood flow leads to erection in men and clitoral engorgement and vaginal lubrication in women. It is of utmost importance to note that the visceral responses are automatic, outside of control by volition [14,46]. It could be stated that the intensity of these unconsciously initiated responses is an exquisite indicator of the level of activity in the central motive state, uncontaminated by conscious manipulations [12,13,47].

The incentive motivation model has become dominant in contemporary research on sexual motivation [48,49,50]. One of the many advantages of this model is that it is and has been used in studies of sexual interaction in humans as well as in non-human animals. Furthermore, different from other models, simple operational definitions of sexual motivation can be proposed, and quantification of the intensity of motivation can be assured by objective measurement rather than by self-reports. Because of these and other reasons (see [47] for an extensive discussion), the incentive motivation model seems to be particularly useful for sex comparisons of the intensity of sexual motivation.

### 2.5. Other Models

There are many additional models or descriptions of the dynamics of sexual interaction between humans, such as the information processing model [46], Basson’s circular model [51,52], and the dual control model [53,54], just to mention a few. None of these models has inspired studies of sex differences. Furthermore, they are not applicable to non-human animals. For these reasons, we do not further discuss them.

## 3. Is Sexual Motivation a Trait or a State?

The incentive motivation model outlined above posits that sexual motivation is absent unless a sexual incentive has activated it. In non-human animals, the incentive stimulus usually originates in a conspecific of the other sex whereas in humans, mental representations (fantasies) of sexually relevant stimuli may function as sexual incentives. A consequence of the need for an incentive for being active is that sexual motivation must be evaluated in the presence of an incentive. This is always carried out in studies of sexual motivation in non-human animals. In humans, however, sexual motivation is frequently evaluated in the absence of incentive stimuli. In fact, an overwhelming majority of the questionnaires employed in clinical practice as well as in research for quantifying sexual desire are based on the assumption that sexual motivation is a stable trait, meaning that it can be evaluated in any context. Following the general acceptance of the incentive motivation model, it has become necessary to distinguish sexual motivation evaluated in non-sexual contexts from the motivation manifested in response to sexual incentives. This quite obvious fact has prompted the use of concepts such as “state desire” vs. “trait desire”. State desire refers to the momentaneous level of desire manifest in the immediate context, generally containing a sexual incentive, whereas trait desire refers to some hypothetical, context-independent level of desire (see, e.g., [55]). State desire is also called responsive desire, to make clear that it is triggered by a sexual stimulus of some kind [56,57].

It may be proposed that trait desire represents the reactivity or responsiveness of the central motive state in the way that high trait desire corresponds to a high level of reactivity to sexual stimuli and vice versa. Studies in humans have indeed provided experimental evidence for the notion of trait desire as a determinant of the responsivity to sexual incentives. For example, the level of trait desire has been found to partly determine the amount of attention paid to sexual stimuli in young women and men [58]. Furthermore, the magnitude of the acoustic startle response and prepulse inhibition during exposure to sexual pictures were related to the level of trait desire [59]. It has also been reported that the whole brain hemodynamic response to sexual stimuli is related to trait desire both in men and women [60]. All these data suggest that the impact of sexual stimuli is determined by the level of trait desire. If this indeed were the case, then trait- and state desire should be correlated, meaning that measurements of trait desire are good predictors of state desire. Perhaps questionnaires measuring state desire fail to provide any meaningful information not already given by estimations of trait desire. Thus, the distinction between state and trait has little merit, and may even obscure our understanding of the mysteries of sexual motivation. It can certainly not be used as an argument neither for nor against the existence of sex differences in the intensity of sexual motivation. 

It is not only with regard to sexual motivation that the distinction between state and trait has attracted attention. In fact, most characteristics of an individual can be conceived either as traits or states [61]. Whole trait theory [62] postulates that states are momentary manifestations of traits. Consequently, the average state (an aggregate of several measurements at different time points) could provide a more accurate and reliable measure of personality characteristics (traits) than global self-reports. This may be the case (e.g., [63,64]), but it has also been proposed that traits and average states are homomorphic, i.e., they are expressions of the same construct [65,66,67]. If so, the distinction between trait and state has little merit. In the following, we will mostly report data obtained during exposure to sexual incentives (state motivation). According to the reasoning exposed above, these data should also be applicable to trait motivation. 

## 4. Quantification of Sexual Motivation in Non-Human Animals and in Humans

A meaningful sex comparison of the intensity of sexual motivation must be based on reliable, quantitative data. Consequently, the procedures used for obtaining these data are of crucial importance. We will now provide a short overview of how the abstract concept of sexual motivation has materialized in measurement techniques. 

### 4.1. Non-Human Animals

**Figure 2:**
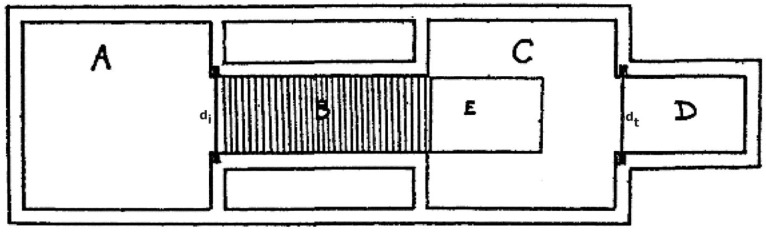
The Columbia obstruction apparatus for study of animal motivation. Diagram of floor plan of the obstruction box. A, entrance compartment; B, obstruction compartment; C, D, divided incentive compartment; E, release plate; di, manually operated door of entrance compartment; dt, automatic door (operated by release plate) between two divisions of incentive compartment. Reprinted from [72]. For a detailed explanation, see the original publication.

It has been suggested that rodent sexual approach behaviors are most appropriate for quantifying sexual motivation [47,68,69,70,71]. In the case of rodents, the intensity of sexual approach behaviors has been extensively used for estimations of sexual motivation. The approach can easily be quantified both in males and females, even with the same experimental setup. The pioneers in the field of sexual motivation used a procedure known as the Columbia Obstruction Box (see Figure 2). If the subject were a male rat, then it had to traverse an electrified grid in order to have access to a sexually active female. If the subject were a female, then it got access to a sexually active male after having crossed the electrified grid.

The obstruction method is a kind of conflict procedure in which the motivation to approach a positive incentive, a mate, is countered by the motivation to withdraw from a negative incentive, the pain inflicted by electric shock. The fact that observed behavior is determined by the sum of opposing motives complicates the interpretation of the results. This complication can be avoided by observing approach behavior in the absence of any aversive element. In fact, it has repeatedly been pointed out that unopposed sexual approach behaviors are exquisite indicators of the intensity of sexual motivation [69,71,73]. 

Even though data from the Columbia Obstruction Box may not be unambiguous measures of sexual motivation, they can at least be regarded as suggestive. Data from procedures evaluating unopposed sexual approaches should be most adequate for sex comparisons.

In other non-human animals, data on sexual approach are rarely available. Researchers have focused on details of copulatory behavior while ignoring that copulation always is preceded by sexual approach. Furthermore, long ago it was pointed out that copulatory behavior is a poor indicator of sexual motivation [71]. There are many parameters of copulatory interactions, and the interpretation of each of them in terms of motivation is far from straightforward [74]. In the absence of direct estimates of sexual approach, approximations need to be made. One such approximation is to determine the number of sexual partners per unit of time when multiple partners are available. The data needed for estimating that number are usually obtained in field studies, having the advantage of being ecologically valid. This is far from the case in most laboratory studies of copulatory interactions.

A more questionable procedure for estimating the intensity of sexual motivation is to count the number of sexual interactions with a single partner per unit time. Since this number depends on the partner as much as on the target individual, it is less informative than the number of different partners. 

### 4.2. Humans

#### 4.2.1. Conscious vs. Unconscious Sexual Motivation

Self-reports are the results of cognitive activities. However, motivation, according to the incentive motivation model used in the present communication, is an initially unconscious process. It is only when an active central motive state manifests itself in genital responses providing conscious sensory feedback or in the experience of sexual desire that consciousness becomes involved. The relationship between the unconscious motive and the conscious answers to items in a questionnaire remains entirely unknown, but it can be expected that these answers are heavily influenced by cognitive elements. In fact, it would have been most surprising if the questionnaire answers had contradicted the script or traditional social learning theories mentioned above since all these theories are based on cognitive processes at a rather high level. There is ample room for altering the unconscious motivation in a way that coincides with acquired expectations and desirable outcomes. Because of this, it is most uncertain if self-reports can offer any useful information regarding the inherent intensity of sexual motivation. What they do is to provide information about how individuals consciously experience their level of sexual motivation, and perhaps also about how the individual wants others to perceive her or his level of that motivation. In search of inherent sex differences, it is necessary to find ways to estimate the intensity of unconscious sexual motivation. 

Even though unconscious mental processes were mentioned already in ancient Greece, it was not until the work of William James and Wilhelm Wundt that such processes were recognized as part of the new science of psychology [75,76]. However, it was the work of Sigmund Freud that turned unconscious mechanisms into the main determinants of human psychic functions [77]. Freud considered all drives as unconscious, and their access to consciousness was strictly controlled by the censor, a mental force preventing the access to consciousness and preventing drives from having any influence upon action tendencies that displease it [78]. The unconscious drives are unable to “bring about any expedient muscular acts, with the exception of those already organized as reflexes” [79] (p. 188). This means that the unconscious motive needs to be allowed into the preconscious and eventually the consciousness before being able to activate volitional motor activities. Other responses to the drives, such as selective attention, genital engorgement, enhanced general arousal, and facilitated spinal reflexes may well be accomplished already by the unconscious drive. In addition to manifesting themselves in these responses, unconscious motives can become accessible through tests for implicit motivation. In the following, we will describe procedures for probing the unconscious dimension of sexual motivation. However, first, we will turn to the subjective experience of that motivation. 

#### 4.2.2. Self-Report Questionnaires

Because so much research on sexual motivation, often called desire in the clinical literature, has been based on self-report questionnaires, it is imperative to give some examples of the items used for estimating the intensity of sexual motivation. Likewise, it is important to illustrate the diversity of assumptions concerning the nature of sexual motivation underlying the choice of items. Some questionnaires emphasize what Moll [17] called the drive for contrectation, sexual approach behaviors in incentive motivation models, while others concentrate on the drive for detumescence and copulatory responses in the incentive model. Still others mix the two kinds of responses.

Already in 2004, 57 questionnaires measuring different aspects of sexual function and dysfunction were identified, including sexual motivation [80]. This number has grown considerably since then [81], and the number of questionnaires evaluating aspects of sexuality is large indeed. An overview of relevant questionnaires would go far beyond the scope of the present contribution. We will only present a few examples.

One of the most used questionnaires is the Sexual Desire Inventory [82]. The authors offer a definition of sexual motivation, or sexual desire as they prefer to call it: “Sexual desire refers to *interest in sexual activity*” [82] (p. 178). They correctly point out that sexual desire is not a behavior, and that it should not be measured based on the occurrence of sexual behaviors such as masturbation and intercourse. One of the 8 items in their dyadic sexual desire scale is “When you have sexual thoughts how strong is your desire to engage in sexual activity with a partner?”. The answer to this item is to be provided on a Likert scale from 1 (no desire) to 8 (strong desire). Another item is “During the last month, how often would you have liked to engage in sexual activity with a partner (for example, touching each other’s genitals, giving or receiving oral stimulation, intercourse, etc.)”. The 7 answer options range from “not at all” to “more than once a day”. Solitary sexual desire is evaluated with 3 items, one of which is “during the last month, how often would you have liked to behave sexually by yourself (for example, masturbating, touching your genitals, etc.)?” Another is “How strong is your desire to engage in sexual behavior by yourself?”. The response alternatives to these items are the same as those in the dyadic desire scale. 

The distinction between desire and actual sexual behaviors was first proposed by Moll [17] and later by the incentive motivation model and preserved by Spector [82] upheld in many questionnaires (e.g., [83]), although others include items concerning the frequency of orgasm or of masturbation (e.g., [84,85]). To the latter group belongs the Frankenbach et al. metanalytic study [4]. It was based on selected items from a total of 16 self-report questionnaires, some referring to the frequency of sexual behaviors and some referring to the intensity of sexual motivation. The choice of items was based on the authors’ conceptualization of sex drive as the “density distribution of state sex drive, where state sex drive is defined as momentary sexual motivation that manifests in sexual cognition, affect, and behavior” [4] (p. 621). It is difficult to determine the exact consequences of the diversity of the theoretical underpinnings of the many self-report questionnaires, but it may contribute to some confusion. Despite the uncertainty with regard to the theoretical bases for most of the many self-report-based questionnaires, they have been extensively used for sex comparisons of the intensity of sexual motivation. In fact, data from this kind of questionnaire constitute the only evidence for a difference between males and females, as mentioned already in the Introduction. The frequent failure to distinguish between sexual approach (contrectation) and copulatory behaviors (detumescence drive) when estimating the intensity of sexual motivation further weakens the conceptual basis for assuming meaningful sex differences. 

It is reasonable to suppose that verbal self-reports either of sexual desire or of past sexual events are limited to conscious experiences. In addition to the many sources of erroneous reports already mentioned, for example, retrospective bias, recall bias, and hosts of other factors (see [86,87] for reviews), the exclusion of unconscious elements considerably reduces the value of self-report data. The fact that the unconscious component is lacking has not been of any concern, perhaps because many researchers make the unwarranted supposition that human sexual motivation is entirely conscious.

Because of the reasons exposed above, we do not consider self-report questionnaires to be appropriate for evaluating sex differences in sexual motivation. Self-report data can perhaps be valuable for determining how humans experience their own sexual behavior, their beliefs concerning the motivation for engaging in that behavior, and perhaps even for inferring the level of satisfaction obtained from sexual acts [88]. All these elements are conscious and therefore accessible for self-reporting. As a difference, the responsivity to sexual stimuli, i.e., sexual motivation according to the incentive motivation models outlined above, is not an entirely conscious event. Therefore, it is not suitable for self-reporting. Other means must be sought to gain access also to the important unconscious component. This proposal does not in the slightest question the clinical value of self-report questionnaires. In fact, they were designed for clinical evaluations and not directly for research purposes.

#### 4.2.3. Tests for Implicit (Unconscious) Sexual Motivation

The Thematic Apperception Test was developed as a means to gain access to unconscious motives [89], and eventually to allow for reliable quantification of their intensity (e.g., [90]). Briefly, subjects are asked to write stories based on ambiguous pictures. This procedure is now known as the picture story exercise (e.g., [91]). A version adapted to the quantification of sexual motivation has been developed recently. Here, the pictures show a social interaction that may lead to sexual interaction but does not necessarily do so. Before the writing tasks, subjects may be exposed either to sexually arousing pictures [92] or to pornographic video fragments [93]. Both kinds of priming stimuli enhanced the number of sexual allusions in the stories, hence implicit sexual motivation, exactly as would be predicted from incentive motivation theory. Further data concerning the reliability and validity of this test for quantifying sexual motivation are found in a book chapter [94].

A far more complex procedure for estimating implicit sexual motivation was described a few years ago, labeled the implicit AMORE (Affective and Motivational Orientation Related to Erotic Arousal Questionnaire) scale [95]. Pictures of female—male couples engaged in sexual activities were presented for a short time, followed by an ambiguous object (a character from the Chinese language). The subjects were asked to indicate whether the second object was pleasant or unpleasant. The data thus obtained were subjected to a series of analyses, and the author concluded that the test is a reliable and valid estimator of implicit sexual motivation. There is also a scale based on the same 8 motivational dimensions as in the implicit AMORE, but employing self-reports [96]. Consequently, that scale measures explicit (conscious) sexual motivation.

The Implicit Association Test [97] detects unconscious associations between concepts and attributes. It is probably the most used test for estimating implicit motivation. The original test has been simplified [98], and the simplified version has been adapted to evaluate implicit sexual motivation [99]. Basically, it consists of determining the strength of mental associations between a concept, for example, attractive or exciting, and an evaluative dimension, in this case sexually desirable vs. sexually undesirable.

Since the measures of implicit sexual motivation focus on the unconscious part, they should be appropriate for making sex comparisons. 

#### 4.2.4. Subliminal Priming

A couple of studies have tried to determine how brief exposure to a sexual stimulus affects subsequent responses to sexual or non-sexual stimuli. It is believed that short exposure times (500 ms or less) prevent a stimulus from reaching consciousness while preconscious processing may be complete. The reasoning behind and the mechanisms involved in subliminal priming have been reviewed in some detail [100]. This procedure has sometimes been used to determine the influence of unconscious processes on variables related to sexual motivation. It seems appropriate for evaluating potential sex differences.

#### 4.2.5. Attention to Sexual Incentives and Cognitive Processing

The first of the events in the sequence leading to sexual activity is the detection of a stimulus followed by the identification of the stimulus as a sexual incentive. Only after this identification, the stimulus may have an impact on the sexual central motive state. The processes of detection and identification are heavily dependent on attentional mechanisms [101,102]. Moreover, it is generally accepted that initial attention and early processing of sexual stimuli are events not reaching the conscience [103]. Provided that information processing is sensitive to variations in general arousal [104] and affected by the level of sexual motivation (e.g., [105], reviewed in [106]), sex comparisons based on data from studies of attentional mechanisms should be most revealing, and free from any bias.

#### 4.2.6. Genital Responses

In non-human animals, visceral responses to sexual incentive stimuli have not been used for estimations of the intensity of sexual motivation. One reason may be that behavioral observations are more convenient. Such observations can be performed without interfering with the animals whereas the recording of visceral responses usually requires sophisticated, invasive procedures. One example could be the telemetric recording of the dynamics of *in copula* intracavernous pressure [107]. This procedure requires surgery for the chronic implantation of pressure transducers, and it is quite costly both in terms of time and equipment. Although offering invaluable data, this procedure is entirely inadequate for routine use. Moreover, invasive procedures always raise concerns about animal welfare [108], and their use is being minimized. Finally, the behavior of non-human animals is not altered by volitional or other conscious processes. It can be considered a true manifestation of the activity in the central motive state. This fact strongly reduces the need for recurring visceral responses. In humans, the situation is entirely different, as mentioned, and visceral responses are one of the few unbiased manifestations of the activity of the sexual central motive state.

In men, the magnitude of the genital response to sexual incentives can easily be recorded as changes in penile circumference, usually determined with a strain gauge transducer. Sometimes penile volume rather than circumference is determined. In fact, the former procedure is superior when the response magnitude is small [109]. In women, vaginal blood volume can be estimated in several ways, but the most common is photopletysmography. The difference in light reflection between systole and diastole, known as vaginal pulse amplitude, is considered to be an exquisite indicator of the vaginal response to a sexual stimulus, hence the intensity of sexual motivation [110]. 

Instead of estimating vaginal congestion, vaginal lubrication can be recorded. A consequence of enhanced vaginal blood flow is that intracapillar blood pressure increases, thereby pressing water and other small molecules out of the bloodstream and into the vagina. This transudate is referred to as vaginal lubrication. The amount of lubrication can be estimated [111], and it may be a sensitive indicator of sexual motivation. However, it seems to be less sensitive than the vaginal pulse amplitude for detecting variations in the intensity of sexual motivation [45]. Furthermore, introital lubrication has no equivalence in men, rendering this measure unsuitable for sex comparisons. To the contrary, the vaginal vascular responses are similar to the penile vascular responses in several ways, most importantly with regard to the nervous control [112,113]. Thus, vaginal and penile plethysmography can be considered appropriate for sex comparisons of motivational responses, particularly since the response is unconscious and non-volitional. We propose that it is an unbiased measure of inherent sexual motivation.

#### 4.2.7. Anal Responses

Men and women show contractile responses in pelvic muscles during sexual arousal [114,115]. These contractions are associated with vascular responses. Both events can easily be recorded with an anal probe [116]. It has been proposed that the anal response to sexual stimuli could be used to make sex comparisons. A photopletysmograph could be inserted into the anal canal in women and men, thereby making it possible to use the same instrument in a similar location in men and women. Unfortunately, men failed to respond with larger anal vasocongestion when exposed to sexually relevant stimuli than when exposed to other kinds of emotional stimuli [117]. This observation shows that the “unisex” procedure lacks specificity for sexual stimuli. Consequently, it is of no use for making sex comparisons of the magnitude of response to sexual incentives, and anal responses will not be further mentioned. 

#### 4.2.8. Spinal Reflexes

Activation of the sexual central motive state leads not only to genital responses but also to enhanced general arousal (see [12], for discussion of this issue). The enhanced general arousal has many effects on central nervous function. One of these effects is to modify spinal reflexes. For example, the Achilles reflex has been reported to be sensitive even to small variations in general arousal [118,119]. Since the activity in the sexual central motive state is one of the determinants of general arousal, hence the sensitivity of spinal reflexes, the magnitude of these reflexes produced by a constant stimulus is an indirect measure of the activity in the sexual central motive state. Sensitization of spinal reflexes produced by exposure to a sexual incentive should, therefore, be a measure of the activity in the central motive state uncontaminated by conscious manipulation. It can, then, be considered an appropriate measure for use in comparisons between men and women.

#### 4.2.9. Frequency of Masturbation in Infants

Even though we have suggested that copulatory behaviors are inappropriate for estimating the intensity of sexual motivation, it is possible that the frequency of infantile masturbation may be an unbiased measure. We will present arguments in favor of this notion in a later section.

## 5. Sex Comparisons

In the following sections, we will first summarize rodent data relevant to sex comparisons, followed by a discussion of non-human primates. Finally, we will turn to the human data. 

### 5.1. Sexual Motivation in Male and Female Rodents

When males and females were compared in the Columbia Obstruction Box, it was found that females were more likely to traverse the electrified grid than males were [120]. It was concluded that female rats have a larger sex drive than male rats. In another experiment, the number of grid crossings per unit time was used as an estimator of the intensity of sexual motivation. The results revealed that the females made more crossings than the males, again showing that females have a more intense sexual motivation than males [121]. A similar observation was reported shortly after [72]. These carefully conducted studies, comparing males and females in identical procedures and conditions, provide suggestive evidence that females are more sexually motivated than males. An extensive review of the early motivation literature repeated this conclusion [122]. However, one study employing the obstruction box failed to detect any sex difference [123]. Nevertheless, it is noteworthy that male rats never have been reported to have larger sexual motivation than female rats.

**Figure 3:**
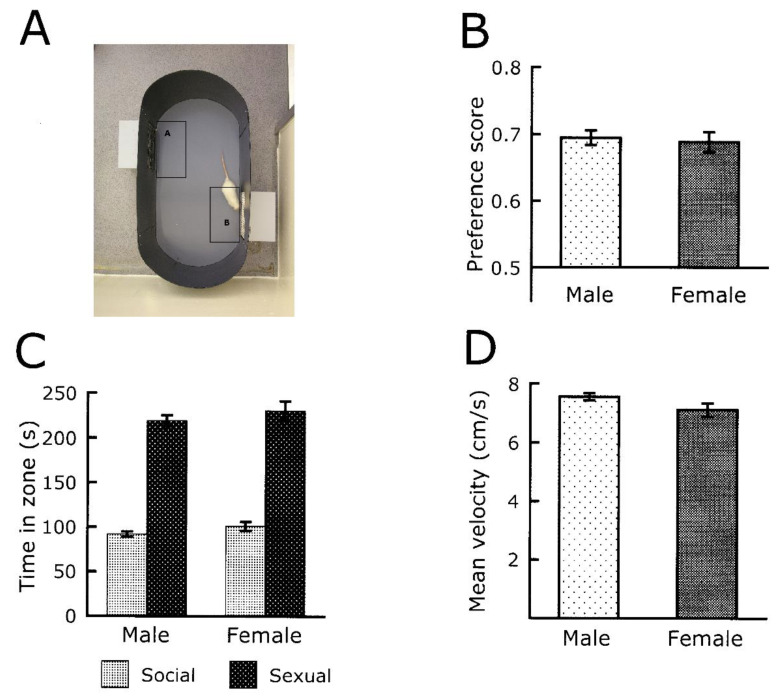
Sexual approach behaviors in sexually active, intact male rats and in sexually receptive, hormone-primed ovariectomized rats. The females had been given estradiol benzoate, 25 µg/rat, 48 h before tests, and progesterone, 1 mg/rat, 4 h before. This treatment assures a maximum level of sexual activity. (**A**) The setup used for quantifying sexual approach behaviors. The rectangles marked A and B delimited the incentive zones. (**B**) The preference score (time spent in the zone outside the sexual incentive/(that time + the time spent in the zone outside the social incentive)) was obtained during a 10 min test. For female subjects, the sexual incentive was a sexually active male, and the social incentive was a castrated male. For male subjects, the sexual incentive was a sexually receptive female, and the social incentive was another, intact male. There was no difference in score between male and female subjects (*t*_134_ = 0.314, *p* = 0.754). (**C**) The time spent in the zone outside the sexual and the social incentives in male and female subjects. ANOVA showed a main effect of incentive (*F*_1,134_ = 297.89, *p* < 0.001, $\eta_{p}^{2}$ = 0.690). The time spent near the sexual incentive was far above that spent in the vicinity of the social incentive. There was no effect of sex (*F*_1,134_ = 3.73, *p* = 0.055, $\eta_{p}^{2}$ = 0.027). Even though the sex difference was close to significance, the small effect size shows that it cannot be functionally meaningful. There was an interaction incentive x sex (*F*_1,134_ = 0.029, *p* = 0.864, $\eta_{p}^{2}$ = 0.000), showing that the superiority of the sexual incentive was equal in males and females. (**D**) Speed of movement while moving provides an excellent estimate of the level of locomotor activity. There was no significant sex difference in the activity recorded during the test (*t*_122_ = 1.836, *p* = 0.069, Cohen’s *d* = 0.370). Activity data from 12 females were lost because of a technical failure, but the remaining 34 should offer an acceptable approximation. Data are mean ± SEM.

The many studies evaluating sexual approach behaviors in procedures without aversive elements have ignored differences. In view of the lack of published data, we have analyzed unpublished data from the Ågmo laboratory. For several years, we have used a simple and reliable procedure for quantifying sexual approach behaviors [68,70]. Hundreds of males and females from the same provider, and kept under identical conditions, have been tested in the same setup, sometimes even on the same day. We have located data from 90 males and 46 females coinciding with this description. Figure 3 illustrates that none of the indices of sexual motivation differs between males and females. Not even a simple measure of ambulatory activity reveals any sex difference. These data suggest that the intensity of sexual motivation is similar in male and female rats. It must be noted that all females were ovariectomized and sequentially treated with supraphysiological doses of estradiol and progesterone before tests. Females treated in this way are indistinguishable from intact females in estrus [68].

Whereas female rats generally showed signs of having more intense sexual motivation than male rats in the obstruction procedure, no sex difference emerges in studies of sexual approach in procedures lacking aversive components. It may be prudent to conclude that the experimental evidence either suggests that there is no sex difference with regard to sexual motivation in rats, or that females are somewhat more motivated than males. We have been unable to find relevant data for the evaluation of sex differences in sexual approach behavior from other rodent species. This summary of available rodent data shows that there is not any consistent experimental evidence for any sex difference at all with regard to sexual motivation.

### 5.2. Sexual Motivation in Non-Human Primates

There are no observational or experimental data that can be used for direct comparisons of the intensity of sexual motivation in male and female non-human primates. There is a large number of studies of primate sexual behavior, both in captivity and in the wild (e.g., [124,125]), but these studies are not directly relevant for estimating the intensity of sexual motivation. We know of only one paper [126] in which the quantification of sexual motivation was the main issue. Erection, rated on a three-point scale using visual observation, was found to be a good measure. The disadvantage is that responses determined by genital blood flow cannot easily be obtained from female primates, thereby precluding sex comparisons.

Above we proposed that the number of sexual partners per unit time can be used as an indicator of sexual motivation. Determinations of these numbers require that there are several potential partners available during the observation period. Even though this could easily be arranged in the laboratory, no such studies have been published. However, observations in the wild are normally performed on groups of animals in which several partners are available simultaneously, making it feasible to record the number of sexual partners per unit time. This has been carried out in some studies. In the gray mouse lemur (*Microcebus murinus*), observed in the Kirindy Forest on Madagascar, females may copulate up to 11 times with 7 different males during a single night of estrus. Median number of copulations as well as of partners was 3 [127]. Similar data for males of this species are not available, but there are observations suggesting that they are not as active as females (e.g., [128]). 

In chimpanzees (*Pan troglodytes*), also observed in the wild, females attempt to mate with all males in the group during the period of sexual swelling [129], and the males try to copulate with all females with such swellings [130]. Thus, male and female chimpanzees are completely promiscuous, copulating with all available partners. This may suggest that the level of sexual motivation is not entirely different in males and females. This is further supported by analyses of the rate of copulation in female chimpanzees living in large groups with several males in the Kibale National Park in Uganda [131]. The females copulated at an average rate of 3–5 times per hour during the period of maximal swelling of the sexual skin. Some female chimpanzees seem to have a sexual motivation of rather high intensity. One female was found to copulate 65 times, with 17 different males, during an observation period of 11 h [131]. Studies of male chimpanzees suggest that the rate of copulation is not superior to that found in females [132]. However, in the absence of studies specifically designed to evaluate sex differences in chimpanzee sexual motivation, this conclusion must be considered as preliminary. Finally, observations of sexual behavior in wild bonobos *(Pan paniscus*) do not offer any evidence for a clear sex difference in the motivation to engage in copulation (reviewed in [133]). 

The examples given here should be enough to show that there is no clearly observable difference in sexual activity between males and females. It can be suggested that males and females in at least some species of non-human primates are equally motivated to engage in sex. In that way, non-human primates may be similar to rodents. Unfortunately, the lack of studies directly evaluating sex differences in the intensity of sexual approach behaviors makes this proposal tentative. 

As was the case with rodents, it must be mentioned that the examples of non-human primate sex behavior mentioned here refer exclusively to the period of the menstrual cycle when the females show maximal receptivity and are maximally attractive to males. This period usually coincides with maximal swelling of the sexual skin, and with ovulation (e.g., [134]). The timing of the primate observations in relation to the cycle phase is of importance, since females in some species are similar to rodents in the way that sexual activity is limited to a specific period of the menstrual cycle. Likewise, some species are seasonal breeders [135], not displaying any activity outside the breeding season. All the examples mentioned here refer to periods of high female sexual activity. In other primate species, females are sexually active regardless of the phase of the cycle or of the season (reviewed in [136]). Then the timing of observation is less important. Obviously, sex comparisons are meaningful only if made during periods in which both sexes are sexually active.

### 5.3. Sexual Motivation in Humans

#### 5.3.1. Implicit Motivation 

The recent modification of the Thematic Apperception Test applied to the measurement of sexual motivation [94] offers preliminary data with regard to implicit sexual motivation. These data are particularly interesting because they come from a study in which the participants were exposed to a sexual incentive (a fragment of a pornographic video showing oral sex and penile-vaginal intercourse) or a neutral stimulus immediately before the picture story writing task. The sexual incentive enhanced implicit sexual motivation, and there was no difference between men and women. Self-reports of sexual motivation replicated the standard finding that men are superior to women [93]. These observations support the notion that sexual motivation is of equal intensity in men and women unless manipulated by volitional processes.

A study employing the implicit as well as the explicit AMORE scales failed to detect any sex difference on the implicit scale, evaluating aspects of unconscious sexual motivation, whereas men scored higher than women on the explicit AMORE scale [95]. These data are interesting because both scales are based on the same theoretical conception of sexual motivation and are consequently comparable. Similar results were reported in another study comparing explicit and implicit sexual motivation [137]. Here, implicit motivation was measured with a variant of the Implicit Association Test [97], and explicit motivation was determined by self-reports of sexual arousal. When implicit motivation was determined, there was no difference between men and women. However, when self-reports were used, men scored higher than women. Finally, an online study of implicit sexual motivation within stable, cohabitating heterosexual couples found no difference in the intensity of implicit motivation between the male and female partners [99]. This study is remarkable because no sex difference was found in explicit sexual motivation, assessed by a slightly modified Sexual Desire Inventory [82]. Among possible explanations for the lack of sex difference may be the questionable reliability and validity of online studies ([138] and references therein). Another explanation may be that the participants felt no conscious need to alter the self-report because of the anonymity of internet studies. 

The finding that there is no sex difference with regard to implicit sexual motivation is consistent over studies. We conclude that data from studies of implicit sexual motivation indicate the absence of any inherent sex difference. 

#### 5.3.2. Effects of Subliminal Priming 

There are few experiments in which the subliminal priming procedure has been employed for evaluating sex differences. In one, men and women were subjected to a conditioning procedure, in which the conditioned stimulus was presented either subliminally (30 ms) or supraliminally (10 s) immediately before the unconditioned stimulus, a 30 s pornographic video fragment. The unconditioned response was genital engorgement. After 11 conditioning trials, a test was made. It turned out that both the sub- and supraliminal stimuli enhanced genital engorgement at the test, showing that conditioning with an unconscious stimulus is feasible. Of particular importance is the fact that there was no sex difference [139]. The level of motivation is one of the determinants of the speed of acquisition of a learned response. Therefore, these data suggest that the level of sexual motivation is similar in women and men. 

In another study, young men and women were subjected to a brief (30 ms) exposure either to pictures of naked individuals of the preferred sex or neutral pictures immediately before performing various tasks or answering questionnaires. It was found that the sexual picture enhanced positive affect and increased motivation to persist on an uninteresting task. More importantly in the present context, the sexual priming also made the subjects more interested in sexual interaction. This latter effect was found in an ingenious procedure, in which the experimental subject was offered the choice between two gifts as a reward for their participation in the study. The gifts were a pen, a useful object for students, or a condom, also useful in some contexts. The subjects primed with the sexual picture chose the condom more frequently than subjects exposed to the neutral picture, suggesting that their momentaneous interest in having sex had been increased by the sexual incentive [140]. The effects of priming were equal in men and women in all tasks. It can be concluded that sexual motivation is of similar intensity in both sexes, since the unconscious exposure to a sexual incentive produced effects of similar magnitude.

Although it may be risky to base any conclusion on only two studies, we suggest that data on the effects of subliminal (unconscious) priming show that unconscious processes indeed are important for sexual motivation and that there is no sex difference. 

#### 5.3.3. Attention to Sexual Incentives and Cognitive Processing

Experimental studies of sex differences in a picture recognition task found that pictures with sexual content were identified faster and more accurately than pictures with neutral content. Men and women performed equally well on this task [141], suggesting that selective attention to sexual stimuli is similar in both sexes. It is likely that early automatic or unconscious processes were involved in the effects found in these studies.

Sexual stimuli are known to induce pupil dilation [142]. As mentioned earlier, this response may not be specific but rather represents general arousal. Nevertheless, it has been reported that the early (<500 ms) pupillary response to pornographic pictures was similar in men and women, whereas later responses showed the traditional sex difference. The latter was also the case for other slow responses such as heart rate and the galvanic skin response [143]. These results may be an example of the lack of sex differences in the unconscious processing of sexual stimuli. As soon as consciousness gets involved in the interpretation of the stimuli and the ensuing response, sex differences appear.

An eye-tracking study measured the duration of fixation on different body regions in pictures of couples engaged in various sexual activities, including oral as well as penetrative sex. Young men and women were similar with regard to the duration of fixation on the genital area [144]. When the pupil diameter was recorded during exposure to video fragments illustrating non-consensual sex, young women and men responded equally [145]. This applies both to the initial and sustained pupillary response. Another study reporting pupil responses to pornographic pictures failed to detect any difference between men and women, regardless of whether the initial (first 1000 ms) or the late (1000–3000 ms) response was analyzed [146]. The lack of difference between early and late response suggests that conscious processes did not alter the pupillary response. 

All these data coincide in suggesting that the attention to and processing of sexual stimuli are similar in men and women. Unfortunately, and as already mentioned, the pupil response is not specific to sexually relevant stimuli. Any arousal-enhancing event can cause pupil dilation. Furthermore, in the experiments mentioned here, late processing was involved, giving room for conscious manipulation of responses. Thus, the data on pupil responses cannot be interpreted as an unambiguous manifestation of inherent sexual motivation, whereas the duration of gaze fixation on genitals probably can.

The importance of conscious alterations of responses is illustrated in an ingenious eye-tracking study in which the hypothesis that women differ from men because of social expectations was explicitly tested. Eye movements were recorded during exposure to sexually relevant pictures under two conditions: Either the participants were aware that their eye movements were recorded, or they were not aware of this fact. All participants ignored the real purpose of the study. The women who knew that their eye movements were tracked made significantly fewer fixations on sexually explicit pictures than the men did, whereas no such difference was observed when the participants were unaware of being tracked [7]. The authors suggest that women knowing that their gaze was recorded avoided looking at the sexually explicit pictures. The risk of being revealed as showing a behavior unfit for females made these women inhibit their natural gaze pattern. This elegant study shows that social desirability and sexual stereotypes influence behavior far beyond the filling out of questionnaires.

The role of sexual preferences for attention to sexual stimuli has also been determined in eye-tacking studies. An experiment in heterosexual men and women employed stimuli of different intensities, the lowest being moving pictures of nude men and women engaged in solitary exercise. The next level of intensity was moving pictures of masturbation. Sexual intercourse between two men, two women, or a man and a woman constituted the highest stimulus intensity. It was found that men and women responded more to the preferred than to the non-preferred stimulus of low intensity, i.e., men attended more to the female stimulus than to the male, while women did the opposite. Men continued to direct their attention selectively toward the female stimulus even in the high-intensity conditions, whereas women equally attended the male and female stimulus of high intensity. Thus, men systematically show response selectivity in agreement with their sexual preferences, whereas women show such selectivity only for stimuli of low intensity [147]. 

One possible explanation for the lack of selectivity of women’s responses is that the sexual central motive state in women is more responsive than that in men, thereby reacting also to non-preferred stimuli. A more responsive central motive state means a higher level of sexual motivation which in turn means that the range of stimuli able to activate sexual responses is wider. Concordant with the notion of a more responsive central motive state in women is the observation that suboptimal sexual incentives indeed produce a genital response in women but not in men. In a creative study, a film showing repeated penile-vaginal intercourse in bonobos was used as a sexual incentive, in addition to the traditional video depicting a man and a woman engaged in this activity [148]. It was found that women responded with enhanced vaginal pulse amplitude to the bonobo stimulus, whereas men did not. Interestingly, neither women nor men reported an increase in subjective sexual arousal in response to the bonobos. Here we have another example of a difference between the genital (unconscious) response to a sexual incentive and the volitional response. The fact that sexual responses are activated by a wider range of stimuli in women than in men may be interpreted as evidence for a higher level of sexual motivation in the former.

**Figure 4:**
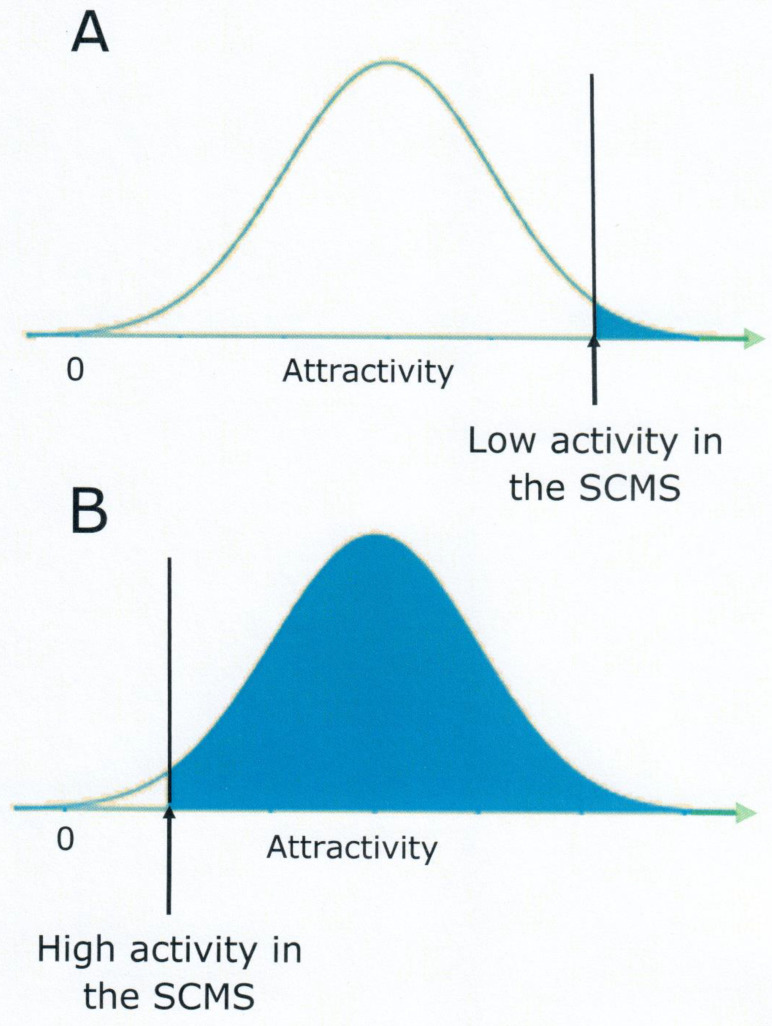
Illustration of the effect of the basic activity of the sexual central motive state (SCMS) on the choice of mating partner. It can be assumed that sexual incentive value, or sexual attractivity, is normally distributed, as shown in the figure. (**A**) The activity in the SCMS is low. Only stimuli with very high incentive value, i.e., stimuli produced by highly attractive individuals, are able to activate the SCMS to the degree that genital responses and eventually sexual approach are stimulated. In the example illustrated here, only 2% of potential mates have such a high level of attractivity. The individual is very choosy. (**B**) Basic activity in the SCMS is high. Even stimuli with low incentive value are able to activate the SCMS, hence sexual approach and visceral responses. As much as 98% of the potential mates have an attractivity level above threshold. The individual shows low, if any, choosiness.

The role of activity in the sexual central motive state and the range of effective stimuli is shown in Figure 4. It is easily seen that there is a direct, positive relationship between these two variables. Even if this explanation for the lack of selectivity in women’s attentional responses to sexual stimuli should turn out to be false, it is difficult to sustain that the lack of selectivity is suggestive of low levels of sexual motivation.

Neither the data on attention to sexual stimuli nor those concerning the selectivity of sexual responses support the notion of any male superiority with regard to the inherent intensity of sexual motivation. Only when volitional alterations of response were possible did a sex difference emerge.

#### 5.3.4. Genital Responses

An early study comparing genital responses to sexual stimuli in men and women was performed by Julia Heiman more than 40 years ago [149]. The results clearly demonstrated that genital arousal patterns in response to erotic audiotapes and sexual fantasies were similar in young men and women. This observation has been replicated many times (e.g., [14,42,150,151]). It seems rather safe to conclude that if there are any sex differences in the genital response to sexual incentives, it is not detectable with available methods. 

While the ease of activation of genital responses as well as the speed of response seem to be similar in men and women, there is a sex difference with regard to the optimal stimulus for provoking a response. In women having men as preferred sex partners, moving pictures of sexual activity with only female participants are equally effective as moving pictures of sex between man and woman with regard to both the vaginal [152] and clitoral responses [153]. In women preferring to have sex with other women, the response seems to be larger to movies showing sex between women than to movies showing a woman having sex with a man [154,155]. Thus, heterosexual women respond equally to movies depicting sex either with the preferred or the non-preferred partner. In women preferring to have sex with other women, the magnitude of the genital response coincides with this preference. In men preferring sex with women, movie fragments depicting sex among men provoke a smaller response than movies showing sex between a man and a woman. On the contrary, in men preferring other men as sex partners, the genital response is larger to a movie portraying sex among men than to a movie illustrating sex between a man and a woman [156,157,158]. Consequently, it is often suggested that men’s genital response is more stimulus-specific than the response in heterosexual women. Ten hypothetical explanations have been put forth for this difference between men and women [159], but none of them has received firm support in experimental data. Nevertheless, it may be appropriate to observe that the lack of specificity in women’s genital response is similar to the lack of specificity found in the studies of attention to sexually relevant stimuli mentioned in the preceding section. We suggested that this lower specificity in women could be attributed to a more responsive sexual central motive state. The same explanation can apply to the genital responses. Indeed, it may be more parsimonious than any of the ten explanations offered in [159].

Not all data coincide with the observations showing low stimulus specificity in women. In the studies showing a lack of specificity of the genital response in heterosexual women, the vaginal or clitoral pulse amplitude was recorded with plethysmography. However, a different picture emerges when another vaginal response, lubrication, is quantified. When the vaginal pulse amplitude and vaginal pH (an indicator of lubrication) were recorded simultaneously in women preferring to have sex with men during exposure to several sexually relevant stimuli in the form of movie segments depicting female—female partnered sex, female—male partnered sex, or male—male partnered sex, the vaginal pulse amplitude increased during exposure to all kinds of sexual incentives whereas lubrication only increased during exposure to the preferred incentive [160]. The female lubrication response showed the same stimulus specificity as the erection response shows in men. 

This short overview of genital responses to sexual stimuli in men and women should have made clear that the dynamics of this response are similar in both sexes. The stimulus control of this response may differ, though. Men systematically limit their response to sexual stimuli depicting sex with their preferred partner whereas heterosexual women may or may not do so, depending on which genital response is recorded. 

#### 5.3.5. Spinal Reflex Responses

In an initial study, young women and men were exposed to different arousal-enhancing events, among those were pornographic movie segments and movies leading to anxiety, while both the Achilles tendon reflex and genital engorgement were monitored. The reflex was elicited with a hammer applying a fixed force for 10 ms to the Achilles tendon, and the response was recorded as an electromyogram from the *gastrochnemius* muscle. The pornographic movie segment but not the anxiety-provoking movie enhanced genital blood flow. However, both movies facilitated spinal reflexes. There was no sex difference in the magnitude of facilitation. The sexual stimulus also activated an action tendency of approach, with similar intensity in both sexes [161]. These observations were interpreted as showing that enhanced arousal facilitates spinal reflexes in preparation for action and that sexual stimuli also activate the intention to approach. In a later study on women, it was found that the more intense the sexual stimuli are, the larger the facilitation of spinal reflexes and, obviously, the larger the genital response [162]. This observation supports the notion that spinal reflex magnitude indeed is related to the level of sexual motivation, reinforcing the conclusion from the first study, that men and women show the same level of motivation after exposure to the same sexual incentives. 

#### 5.3.6. Masturbation in Infants

Tactile stimulation of the genitals produces a state of positive affect in humans and other animals because of prewired connections between the sensory receptors on the sex organs and sites in the central nervous system, as mentioned several times already. These prewired connections are basic for all sexual functions (discussed in [13]). We also mentioned that neutral stimuli may acquire sexual significance through association with the positive effect generated by genital stimulation. Pleasurable genital stimulation in some way or another occurs during the entire lifetime, offering plentiful opportunities for this stimulation to become associated with external stimuli. The fact that the male genitals are protruding from the body surface is commonly interpreted as making them more accessible to mechanical stimulation than the sunken female genitals. Following this astute but starkly androcentric observation, boys and young men are expected to form more frequent associations between pleasurable genital stimulation and all kinds of external stimuli than girls and young women. However, studies show that masturbation is more frequent in female than in male infants [163,164]. Even though these reports are not based on representative samples, they appear to contradict the old belief that male genitals are by nature more accessible for stimulation, hence they also receive more stimulation, than the female genitalia. It may be noted that this belief has, as far as we know, never been substantiated by any empirical data. Rubbing the thighs together or rocking the genital zone against a pillow or another object, common behaviors in masturbating infants [165], may even provide more efficient stimulation of the clitoris than of the penis. It is also interesting to note that the bodily responses during infantile masturbation are similar to those observed at orgasm. In fact, “the parents often recognize the child’s reaction as such” [165] (p. 676). 

Besides the observations of masturbation in male and female infants, the obstetrical literature offers a few reports of masturbation-like activity during the fetal period. A sonographic study not only described clitoral caresses but also a behavior pattern close to that of orgasm in a 32-week-old fetus [166]. Evidence exists for erection and masturbation also in male fetuses [167]. The fact that self-stimulation of the genitals occurs already during the fetal period, together with the observation of orgasm-like motor patterns, could suggest that this kind of stimulation produces positive affects in females as well as in males already before birth. In our societies, characterized by the concealment of sexual matters to children [168] the autoerotic activity of infants is unlikely to be influenced by social learning. 

Questionnaire-based studies, in which mothers answered questions about their child’s sexual activities, have shown that there are no systematic differences between boys and girls aged 2 to 12 years with regard to the frequency of a large range of sexual behaviors [169,170]. However, there was an age-dependent decline in the frequency of these behaviors in both genders, starting from a maximum at about 5 years of age. The authors do not provide any explanation for this decline, but we propose, as a reasonable sociological hypothesis, that when children come to understand that sexual behavior should not be displayed, they simply cease to display it in public or when the parents are looking. To please those attached to the Freudian notion of a latency period, we could also propose that the decline in observable masturbation coincides with the onset of that period.

Different from the preadolescent period, questionnaire studies of adolescent girls and boys show that masturbation is far more common in the latter [171]. At adolescence, the social norm in contemporary Western societies has become clearly differentiated: It coerces feelings of shame in girls and encourages sexual displays in boys [172]. As a result, women experience more negative and less positive emotions related to sex than men, and they also feel decreased sexual autonomy [173]. In fact, the increasing power of the gendered script exerts a growing influence on questionnaire responses as the children develop. This may also affect answers to items in retrospective studies (e.g., [174]) of sex in adolescence, and raise doubts concerning the origin of the asymmetry between boys and girls with regard to adolescent masturbation. Social factors rather than inherent, biologically determined differences, may underly both possible differences in actual behavior and the different responses to questionnaires.

The ensemble of studies mentioned in the preceding paragraphs shows that boys and girls, from infancy until the beginning of adolescence, masturbate with about equal frequency. We suggest that it is likely that there is no difference in the amount of mechanical stimulation of the genitals that boys and girls self-provide or obtain during the course of sexual or non-sexual play. Under the condition that this indeed is the case, both sexes would have the same opportunity to associate the genital pleasure created by mechanical stimulation of the genitals with external stimuli, and the resulting repertoire of stimuli with sexual significance would be equal or close to equal in both sexes. Thus, the anatomical differences between boys’ and girls’ genitals are not necessarily causing differences in psychosexual development. Neither are they necessarily leading to diverging levels of sexual motivation in adulthood. 

### 5.4. Summary of Sex Differences

In Table 1, we have summarized the results of the many studies of sex differences in sexual responses employing methods other than self-reports. Common to these methods is that the recorded response is determined by automatic, unconscious (implicit) processes, inaccessible to volitional control. We have presented arguments suggesting that the magnitude of these responses is controlled by the momentaneous level of sexual motivation. 

## 6. Why Are Men and Women Responding Differently to Questionnaires?

In the Introduction, we mentioned some of the factors that might affect responses in self-report questionnaires. Here we will provide an overview of some of the theoretical approaches that underlie the purported sex difference in sexual motivation. Some of them can become important determinants of how humans report their sexual motivation.

### 6.1. Classical Explanations

In humans, the sexual approach behaviors, the copulatory acts, and what is considered appropriate behavior for each sex are social constructions [175,176]. It is generally accepted that men and women follow pre-established scripts before and during sexual interactions [177] and that expectations concerning appropriate male and female behavior are part of these sexual scripts [178]. According to script theory, men are sexually active, dominant, and initiators of sexual activity, whereas women are expected to be reactive to men’s approaches, but otherwise passive and submissive (e.g., [179], see also [180]). These expectations may be as important as, or perhaps even more important than, the level of sexual motivation as a determinant of sexual behavior. Speculations concerning the relationship between sexual scripts and sexual motivation go far beyond the purpose of this review, but we propose that scripts make women underestimate their sexual motivation when filling out questionnaires. Thus, the apparent sex difference in questionnaire-estimated sexual motivation does not necessarily reflect a similar difference in inherent sexual motivation. It may be of interest to note that similar opinions had been expressed in traditional social learning theory [181]. An excellent description of the dilemmas produced by the experience of sexual desire and the potential conflict with social norms in adolescent girls is found in an extensive sociological study [182]. It illustrates how gendered sexual scripts become important determinants of behavior, and presumably of responses to questionnaires. 

Scripts or social learning theory do not propose any sex difference in the responsivity of the sexual central motive state. Thus, they are compatible with the data reported in Section 5, Sex Comparisons.

Script theory is not the only possible explanation for the difference between men’s and women’s self-reports of sexual matters. Within the psychoanalytic tradition, Nancy Chodorow’s psychoanalytic feminism proposes that women perceive men as erotic objects not requiring emotional commitment for sexual relations, but at the same time, men are regarded as providers. For women, sexual dealings should be limited to committed relationships, for example, marriage, with the purpose of maximizing economic security. The result is that women emphasize non-sexual aspects of relationships, different from men, who are centered on carnal sexuality [183,184]. This means that men express more favorable attitudes toward sex, and also manifest a higher level of sexual motivation. However, this difference is not a result of a corresponding difference in the activity of the sexual central motive state. Chodorow’s analysis of sexual motivation and its expression coincides with the data reviewed in the preceding section.

Sociobiology, or evolutionary psychology as it is often called, employs evolutionary arguments for justifying male superiority in sexual motivation (e.g., [185]). It is believed that the sex difference is innate, a postulate reminiscent of Moll’s [17] and McDougall’s [18] discredited models. According to this view, the sex difference in responses to self-report questionnaires is a result of this inborn difference in sexual motivation, hence in the responsivity of the sexual central motive state. This viewpoint is contrary to all the data reported in Section 5.

Rather than persisting in a list of possible explanations of the sex-dependent answers to questionnaires, we refer the reader to an excellent summary of the many possible explanations for the sex differences in questionnaire responses [186]. Even though this paper is 30 years old, its précis of the different theoretical approaches and the corresponding cause of the sex differences has not been surpassed. It may, however, be convenient to relate the proposals made in that paper to the findings illustrated in Table 1 of the present communication.

### 6.2. The Role of Sexual Experiences

An entirely different explanation for the questionnaire-based sex difference is that women indeed are less sexually motivated than men, but not because of an intrinsic sex difference in motivation. Rather it would be due to the fact that women experience less satisfaction with several sexual activities than men do [187,188]. The proportion of sexual encounters leading to orgasm is far higher in men than in women [189,190], whereas far more women than men experience pain during penile-vaginal intercourse [191,192]. Women are victims of sexual violence far more frequently than men [193,194], and fear of putting oneself in a potentially dangerous situation may restrain women’s desire to have sex with unknown or little-known men [195,196]. Moreover, prior experience with sexual violence reduces sexual motivation [197]. Fear of unwanted pregnancy may also reduce the willingness to perform intercourse and weaken expressions of sexual motivation [198]. Likewise, women are expected to be more reluctant to and less eager to perform sexual activities than men, and women’s manifestations of sexuality may lead to considerable stigma [180]. For these and other reasons (reviewed in [187]), the predicted reward value of sexual activity should be lower in women than in men. According to the incentive motivation model, this would make the conscious desire to engage in such activities lower (see [13], for an extensive discussion of this issue). Thus, inferior quality of sexual experiences in women rather than intrinsically low sexual motivation would cause the sex difference found in questionnaire studies. This proposal coincides with the conclusion of an extensive analysis of gender differences in the opportunity to obtain sexual pleasure. Not only do women experience less pleasure than men, but they also pay a higher cost for having sex. These differences are not biologically determined, but results of culturally imposed restrictions on women’s opportunity for sexual pleasure [199]. 

The importance of sexual reward is illustrated in a recent study showing that the amount of sexual satisfaction experienced during a hook-up was the most powerful determinant of the likelihood of engaging in future hook-ups [200]. The favorable experience obtained enhanced the incentive value of stimuli associated with the hook-up and reinforced predictions of reward, thereby enhancing activity in the sexual central motive state. In the same way, an unsatisfactory experience would reduce the incentive value of relevant stimuli and weaken predictions of favorable consequences [201], thereby reducing activity in the sexual central motive state. 

If bad sexual experiences reduce the incentive value of sexually relevant stimuli, hence the activity in the sexual central motive state, then it might be expected that also the automatic responses to sexual incentives would be reduced. However, this is not the case. Instead, the lack of sex difference in the automatic responses analyzed in the present communication suggests that women’s inferior sex experiences only affect cognitively determined (conscious) responses, such as wanting to have partnered sex. In fact, there are many examples of the independent control of the automatic and cognitively mediated sexual responses. Asexuals, for example, have no desire to engage in partnered sex, while their genital response to sexual incentives is equal to that of sexually active individuals [202]. Likewise, women diagnosed with low sexual interest/arousal disorder show a vaginal response to sexual incentives of the same magnitude as healthy women [203,204,205].

Thus, rather than an inherent sex difference in sexual motivation, the difference in questionnaire-based data may reflect an acquired sex difference caused by women’s inferior sexual experiences. According to these arguments, women do not consciously alter their responses to make them coincide with sexual scripts but respond honestly. Nevertheless, these responses should not be interpreted as evidence of an inherently low sexual motivation.

Before ending this section, it must be pointed out that several studies have shown that women actually report higher levels of sexual satisfaction than men do (e.g., [206,207], reviewed in [208]). Since sexual satisfaction can be conceived in many different ways [88], and since its quantification is not straightforward, inconsistent results are not unexpected. For example, whereas physical satisfaction (orgasm) at sexual debut has consequences for future sexual desire, affective satisfaction does not [209]. Moreover, orgasm consistency is only a marginal predictor of genital arousal and is unrelated to conscious sexual arousal according to recent data [210]. The contradictory results make it risky to attribute the sex difference in self-reported sexual motivation to different levels of sexual satisfaction.

## 7. Conclusions

There is a substantial quantity of data showing that unconscious sexual motivation, either manifested in automatic responses to sexual incentives or revealed in tests for implicit sexual motivation, does not differ between men and women. This coincides with available data from non-human animals, where no sex differences in the intensity of sexual motivation can be discovered when appropriate observation procedures are used. The fact that self-report studies consistently find that women have lower sexual motivation than men can be explained by conscious manipulations of self-perceived motivation according to sexual scripts. Whether differences between women and men in the level of satisfaction produced by sexual acts contribute to differences in self-reported motivation remains unclear.

## Figures and Tables

**Table 1:** Sex differences in some indices of the intensity of sexual motivation in several non-human animals as well as in measures outside of volitional control in humans.

Parameter	Species	Result	References
Proportion of animals crossing grid in the Columbia Obstruction Apparatus	Rat	F > M	[120]
Number of grid crossings in the Columbia Obstruction Apparatus	Rat	F > M	[72,121]
Number of grid crossings in the Columbia Obstruction Apparatus	Rat	F = M	[123]
Number of partners per unit time	Mouse lemur	F = M	[127,128]
Number of partners per unit time	Chimpanzee	F = M	[129,130,131,132]
Number of partners per unit time	Bonobo	F = M	[133]
Identification of sexual pictures	Human	F = M	[141]
Fixation on genitals while observing sex	Human	F = M	[144]
Pupil diameter while watching non-consensual sex	Human	F = M	[145]
Implicit motivation as evaluated with the implicit AMORE test	Human	F = M	[95]
Implicit motivation as evaluated with the Implicit Association Test	Human	F = M	[137]
Implicit motivation as evaluated with the modified Implicit Association Test	Human	F = M	[99]
Enhanced motivation after subliminal priming with sexual incentives	Human	F = M	[139,140]
Identification of sexual pictures	Human	F = M	[141]
Pupil dilation in response to sexual stimuli	Human	F = M	[143,145,146]
Gaze fixation on genital area	Human	F = M	[7,144]
Genital responses to sexual incentives	Human	F = M	[14,42,150,151]
Spinal reflex facilitation by sexual incentives	Human	F = M	[161]
Frequency of masturbation in infants	Human	F > M	[163,164]
Frequency of masturbation in adolescents	Human	F < M	[171]

Data from non-human animals, more specifically from rodents and a few non-human primates, show that there is no systematic sex difference in the level of sexual motivation, regardless of how it was quantified. In humans, objective measures of sexual motivation, such as genital responses to sexual incentive stimuli, attentional resources allotted to the processing of these stimuli, the pupil response to them, or the facilitation of spinal reflexes when exposed to sexual stimuli, fail to find any consistent difference between men and women. Likewise, projective tests for implicit motivation do not offer any support for a sex difference with regard to sexual motivation. This is in sharp contrast to the notorious sex difference when it comes to self-reported sexual motivation and sexual behaviors.

## Data Availability

The data presented in this study are available on request from the corresponding author.
